# Enhancing COVID Rehabilitation with Technology (ECORT): protocol for an open-label, single-site randomized controlled trial evaluating the effectiveness of electronic case management for individuals with persistent COVID-19 symptoms

**DOI:** 10.1186/s13063-022-06578-1

**Published:** 2022-09-02

**Authors:** Simon Hatcher, Joel Werier, Nicole E. Edgar, James Booth, D. William J. Cameron, Vicente Corrales-Medina, Daniel Corsi, Juthaporn Cowan, Pierre Giguère, Mark Kaluzienski, Shawn Marshall, Tiago Mestre, Bryce Mulligan, Heather Orpana, Amanda Pontefract, Darlene Stafford, Kednapa Thavorn, Guy Trudel

**Affiliations:** 1 Clinical Epidemiology Program, Ottawa Hospital Research Institute, 1919 Riverside Drive, Suite 406, Ottawa, ON Canada; 2 Department of Psychiatry, University of Ottawa, 5457-1145 Carling Avenue, Ottawa, ON Canada; 3 Department of Mental Health, The Ottawa Hospital, 501 Smyth Road, Ottawa, ON Canada; 4 Department of Surgery, The Ottawa Hospital, 501 Smyth Road, Ottawa, ON Canada; 5 Ontario Workers Network, The Ottawa Hospital, 501 Smyth Road, Ottawa, ON Canada; 6 Ottawa, Canada; 7 Division of Infectious Diseases, University of Ottawa, 451 Smyth Road, Ottawa, ON Canada; 8 School of Epidemiology and Public Health, University of Ottawa, 600 Peter Morand Crescent, Ottawa, ON Canada; 9 Department of Medicine, University of Ottawa, 501 Smyth Road, Ottawa, ON Canada; 10 Centre of Infection, Immunity, and Inflammation, University of Ottawa, 451 Smyth Road, Ottawa, ON Canada; 11 Department of Pharmacy, The Ottawa Hospital, 501 Smyth Road, Ottawa, ON Canada; 12 School of Pharmaceutical Sciences, University of Ottawa, 451 Smyth Road, Ottawa, ON Canada; 13 Division of Physical Medicine and Rehabilitation, University of Ottawa, 505 Smyth Road, Ottawa, ON Canada; 14 Bruyère Research Institute, 85 Primrose Avenue, Ottawa, ON Canada; 15 Parkinson’s Disease and Movement Disorders Center, Division of Neurology, Department of Medicine, University of Ottawa, 501 Smyth Road, Ottawa, ON Canada; 16 Neuroscience Program, Ottawa Hospital Research Institute, 501 Smyth Road, Ottawa, ON Canada; 17 University of Ottawa Brain and Mind Research Institute, 451 Smyth Road, Ottawa, ON Canada; 18 Department of Psychology, The Ottawa Hospital, 501 Smyth Road, Ottawa, ON Canada; 19 School of Psychology, University of Ottawa, 136 Jean-Jacques Lussier Private, Ottawa, ON Canada; 20 Public Health Agency of Canada, 130 Colonnade Road, Ottawa, ON Canada; 21 Department of Medicine, The Ottawa Hospital, 501 Smyth Road, Ottawa, ON Canada; 22 Department of Biochemistry, Microbiology and Immunology, University of Ottawa, 451 Smyth Road, Ottawa, ON Canada

**Keywords:** COVID-19; SARS-CoV-2; Long COVID, Post-COVID rehabilitation, Blended care, Electronic case management

## Abstract

**Background:**

As of May 2022, Ontario has seen more than 1.3 million cases of COVID-19. While the majority of individuals will recover from infection within 4 weeks, a significant subset experience persistent and often debilitating symptoms, known as “post-COVID syndrome” or “Long COVID.” Those with Long COVID experience a wide array of symptoms, with variable severity, including fatigue, cognitive impairment, and shortness of breath. Further, the prevalence and duration of Long COVID is not clear, nor is there evidence on the best course of rehabilitation for individuals to return to their desired level of function. Previous work with chronic conditions has suggested that the addition of electronic case management (ECM) may help to improve outcomes. These platforms provide enhanced connection with care providers, detailed symptom tracking and goal setting, and access to relevant resources. In this study, our primary aim is to determine if the addition of ECM with health coaching improves Long COVID outcomes at 3 months compared to health coaching alone.

**Methods:**

The trial is an open-label, single-site, randomized controlled trial of ECM with health coaching (ECM+) compared to health coaching alone (HC). Both groups will continue to receive usual care. Participants will be randomized equally to receive health coaching (± ECM) for a period of 8 weeks and a 12-week follow-up. Our primary outcome is the WHO Disability Assessment Scale (WHODAS), 36-item self-report total score. Participants will also complete measures of cognition, fatigue, breathlessness, and mental health. Participants and care providers will be asked to complete a brief qualitative interview at the end of the study to evaluate acceptability and implementation of the intervention.

**Discussion:**

There is currently little evidence about the optimal treatment of Long COVID patients or the use of digital health platforms in this population. The results of this trial could result in rapid, scalable, and personalized care for people with Long COVID which will decrease morbidity after an acute infection. Results from this study will also inform decision making in Long COVID and treatment guidelines at provincial and national levels.

**Trial registration:**

ClinicalTrials.gov NCT05019963. Registered on 25 August 2021.

## Administrative information

Note: The numbers in curly brackets in this protocol refer to Standard Protocol Items: Recommendations for Interventional Trials (SPIRIT) checklist item numbers. The order of the items has been modified to group similar items (see http://www.equator-network.org/reporting-guidelines/spirit-2013-statement-defining-standardprotocol-items-for-clinical-trials/).

**Table:** 

Title {1}	Enhancing COVID Rehabilitation with Technology (ECORT): Protocol for an open-label, single site randomized controlled trial evaluating the effectiveness of electronic case management for individuals with persistent COVID-19 symptoms.
Trial registration {2a and 2b}.	ClinicalTrials.gov NCT05019963
Protocol version {3}	Version 23 Feb 2022
Funding {4}	This study is funded by the Canadian Institutes of Health Research (CIHR). The funders have no role in the design or conduct of the study or the decision to publish results.
Author details {5a}	(1) Clinical Epidemiology Program, Ottawa Hospital Research Institute (2) Department of Psychiatry, University of Ottawa (3) Department of Mental Health, The Ottawa Hospital, (4) Department of Surgery, The Ottawa Hospital (5) Ontario Workers Network, The Ottawa Hospital (6) Ottawa, Canada (7) Division of Infectious Disease, University of Ottawa (8) School of Epidemiology and Public Health, University of Ottawa (9) Department of Medicine, University of Ottawa (10) Centre of Infection, Immunity, and Inflammation, University of Ottawa (11) Department of Pharmacy, The Ottawa Hospital (12) School of Pharmaceutical Sciences, University of Ottawa (13) Division of Physical Medicine and Rehabilitation, University of Ottawa (14) Bruyère Research Institute (15) Parkinson’s Disease and Movement Disorders Center, Division of Neurology, Department of Medicine, The Ottawa Hospital (16) Neuroscience Program, Ottawa Hospital Research Institute (17) University of Ottawa Brain and Mind Research Institute (18) Department of Psychology, The Ottawa Hospital (19) School of Psychology, University of Ottawa (20) Public Health Agency of Canada (21) Department of Medicine, The Ottawa Hospital (22) Department of Biochemistry, Microbiology and Immunology, University of Ottawa
Name and contact information for the trial sponsor {5b}	Trial Sponsor: Ottawa Hospital Research Institute (OHRI) Contact name: Dr. Duncan Stewart Address: 501 Smyth Road, Ottawa, ON K1H 8L6 Telephone: (613) 798-5555, ext.79017 Email: djstewart@ohri.ca
Role of sponsor {5c}	The sponsor (OHRI) had no role in the design of this study and will not have any role during its execution, analyses, interpretation of the data, or decision to submit results.

## Introduction

### Background and rationale {6a}

In March 2020, the World Health Organization (WHO) declared the novel coronavirus (COVID-19) a global pandemic. At the time of writing this protocol in February 2022, Ontario had confirmed more than 628,000 cases of SARS-CoV-2 since testing began. Most recently, estimates in May 2022 show this number has climbed to 1.3 million cases [1]. For most of these patients, symptoms resolve within 4 weeks of onset. However, it is becoming apparent that a significant subset of infected patients are experiencing symptoms that persist long after the typical course of acute viral infection. These individuals experience a wide constellation of symptoms, with variable intensity over time and often varying from initial acute presentation [2–6]. Currently, the prevalence and duration is not clear, nor is there evidence on the best course of rehabilitation for individuals to return to their desired level of function.

#### What is post-COVID syndrome?

The National Institute for Healthcare and Excellence (NICE) defines two distinct groups: ongoing symptomatic COVID (symptoms for 4–12 weeks) and post-COVID syndrome, which describes those with ongoing symptoms, not better explained by another diagnosis, for 12 weeks or longer [7]. The most common symptom experienced is persistent fatigue, followed by dyspnea, cognitive issues such as “brain fog” and cardiothoracic issues [2, 5, 6, 8]. Other symptoms include headaches, muscle and joint pain, persistent cough, and psychiatric conditions [2, 6, 8, 9]. An early study found that nearly half of the sample had also experienced a decrease in their quality of life [6]. It is unclear who is most at risk for developing post-COVID syndrome. While severe illness or long time to acute recovery is predictably associated with poor long-term outcomes, more than 35% of individuals with mild illness failed to return to baseline health status [10]. Women [2, 4] and ethnic minorities [11] may also be at higher risk. Previous research from the SARS pandemic also suggests that social disadvantage, such as job loss or social stigma, were also risk factors for long-term symptoms [12]. The heterogeneous nature of symptoms and risk factors suggest that an “organ-based” or classification approach will not provide optimal outcomes for these patients. An integrated model for rehabilitation, including interdisciplinary care, enhanced case management, and screening tools, should be examined [13, 14].

##### Nomenclature

There are at least four terms employed to describe persistent COVID symptoms, including “Long COVID,” “COVID-19 syndrome,” “post COVID-19 syndrome,” or “post-acute sequelae of SARS-CoV-2 infection” [2]. We have chosen to refer to the condition as Long COVID as this seems to be the term most preferred by patients [15].

#### Case management in COVID-19 rehabilitation

Previous work with chronic conditions has suggested that using digital health platforms can improve outcomes [16, 17]. These platforms usually have a patient facing app and a clinician facing dashboard and allow personalization of information and resources to the user; the tracking of symptoms, often assisted by the smartphone’s biometric sensors; contain educational resources; allow for goal setting and monitoring; facilitate collaborative care; and, create a connection between the patient and their clinician [18]. Although the size of the effect varies by condition and intervention [16, 17], these platforms are often low burden to disseminate and adaptable to individual schedules and may increase access to specialized care to patients across rural and remote regions.

Despite the explosion in e-health and coaching platforms available, a recent systematic review found there were few high-quality trials examining the effectiveness of the intervention or its individual components [19]. These platforms have been used across multiple disorders including diabetes [20–22], mental health [23–25], and cancer recovery [26, 27]. These e-health platforms also offer the opportunity for improved communication between the patient, caregivers, and care providers, increasing both understanding of and adherence to treatment protocols [18, 25, 28, 29]. In the case of Long COVID, the addition of a case management platform may prove particularly beneficial. As information is changing rapidly, patients may have more questions about their health concerns and their treatment plan. With electronic case management, clinicians can readily push new resources and information to patients and offer caregivers a more meaningful role.

#### Rationale

The most comprehensive relevant guideline regarding Long COVID is the NICE “COVID-19 rapid guideline; managing the long-term effects of COVID-19” published in December 2020 [7]. The guideline made key recommendations for research including “What is the clinical effectiveness of different service models of multimodality/multidisciplinary post-COVID-19 syndrome rehabilitation in improving patient-reported outcomes (such as quality of life).” The NICE guideline also recommended that patients use symptom diaries or a tracking app to monitor their goals, recovery, and any changes in their symptoms. This proposal directly addresses this recommendation, as the NICE guidelines provide no research to support this approach [7]. We aim to utilize these recommendations to evaluate what possible service models incorporating electronic case management (ECM) will improve outcomes in this population.

## Objectives {7}

### Primary objective

The primary objective of this trial is to determine if adding ECM with health coaching to usual care improves participant quality of life after 3 months for patients who present to hospital outpatient clinics experiencing Long COVID.

We hypothesize that adding ECM with health coaching to usual care will improve outcomes at 3 months compared to usual care alone.

### Secondary objectives

The key secondary objectives of this study are to determine (1) the cost-effectiveness of adding ECM to usual care for Long COVID patients; (2) what factors predict outcomes at 3 months in patients with Long COVID; (3) if the addition of ECM affects Long COVID symptoms; and (4) how a personalized rehabilitation program supported by a digital platform could be implemented for individuals with Long COVID.

We hypothesize that adding ECM to usual care will be cost-effective from a public-payer’s perspective. We also hypothesize that people with Long COVID who had severe acute COVID infections, adverse childhood experiences, recent negative life events, or a strong sense of injustice at baseline will have poorer outcomes at 3 months. Lastly, we hypothesize that those with ECM will show more improvement across outcome measures at 12 weeks.

Following the Medical Research Council guidance on developing and evaluating complex interventions [30], we will conduct a process evaluation guided by the RE-AIM implementation framework [31].

## Trial design {8}

The proposed study is a 12-month open-label, parallel group, randomized controlled trial. Participants will be allocated 1:1 into either electronic case management (ECM+) with health coaching plus usual care or health coaching plus usual care alone (HC). Due to the nature of the intervention, it will not be possible to blind clinicians or patients to the allocation.

## Methods: participants, interventions, and outcomes

### Study setting {9}

This single-site study will be conducted at the Ottawa Hospital Research Institute (OHRI). Recruitment will occur through two different streams to increase the diversity of participants. The first stream is a clinic that sees Ontario workers who were infected by SARS-CoV-2 at their worksite, who have made a claim to the Ontario Workplace Safety and Insurance Board (WSIB) and are assessed by the Ontario Workers Network (OWN) based at The Ottawa Hospital (TOH). The second stream are clinics within TOH which are seeing patients with Long COVID. These are clinics specifically for people with Long COVID, for example within the rehabilitation service, or specialist clinics that are seeing people with Long COVID such as respirology, infectious diseases, or psychiatry.

### Eligibility criteria {10}

Participants will be recruited from TOH. Participants will be approached by an individual in their circle of care if they meet the eligibility criteria outlined in Table 1.

**Table 1:** Eligibility criteria

Inclusion criteria	
Participants must:	
1.	Be 18 years of age or older
2.	Have a confirmed diagnosis of COVID-19 with a PCR test at least 12 weeks prior OR a confirmed rapid antigen test at least 12 weeks prior OR meet Ottawa Public Health guidance for a suspected COVID-19 case (Table 2)
3.	Have at least one ongoing symptom consistent with Long COVID as measured by the WHO Post COVID Case Report Form (CRF)
4.	Have a minimum WHO Disability Assessment Scale (WHODAS) self-report (36 item) sum score of 15
5.	Be willing to use email for study activities
6.	Be able and willing to use an electronic platform, either on smartphone or computer web browser, for the duration of the trial
7.	Be able to read and understand English or French.
8.	Be willing and able to provide informed consent.
Exclusion criteria	
Participants must not:	
1.	Have any significant functional impairment (for example, advanced dementia, heart or lung disease) as judged by the assessing clinician
2.	Participate in another Long COVID trial where treatment is required in the protocol (pharmacological or behavioral). Observational studies will be allowed.
3.	Have symptoms consistent with Long COVID that are better explained by an alternative diagnosis

#### COVID-19 testing eligibility

Effective December 30, 2021, the government of Ontario significantly restricted the eligibility for PCR testing. The general public is no longer eligible for symptomatic PCR testing (https://news.ontario.ca/en/backgrounder/1001387/updated-eligibility-for-pcr-testing-and-case-and-contact-management-guidance-in-ontario). Rapid Antigen Tests (RAT) are also significantly limited in Ontario and these results are not officially reported to any health agency. Additionally, in early 2020, testing was unavailable or highly limited. A previously approved, but not implemented, version of this protocol limited eligibility to PCR test only. To ensure that we are able to enroll individuals, inclusion criteria for testing will not be limited to PCR. The preferred confirmation of eligibility will remain a PCR test 12 weeks prior. Second line of eligibility confirmation will be a positive RAT 12 weeks prior. Third line will be for the participant to confirm a suspected case of COVID-19 indicating they were experiencing symptoms at least 12 weeks prior. Determination of a suspected case will follow the Ottawa Public Health Guidelines (https://www.ottawapublichealth.ca/en/public-health-topics/resources/Documents/Self-Isolation-Flowchart_EN.pdf) summarized in Table 2.

**Table 2:** Suspected COVID-19 eligibility criteria

If you experienced one of:	If you experienced two or more of:
Fever/chills Cough Shortness of breath Decrease/loss of smell or taste	Sore throat Headache Runny nose/nasal congestion Extreme fatigue Muscle/joint pains GI symptoms (vomiting, diarrhea)

Potential participants will be asked to confirm that symptoms were a minimum of 12 weeks ago, and the delegated research staff will document the date and the list of symptoms experienced. The Principal Investigator will then make the final determination about inclusion in the study.

#### Access to technology

Participants are not required to have a smartphone with a data plan in order to participate in the intervention arm. Participants will be able to access their patient dashboard from their computer or tablet with internet access. Participants who do not have any available options to access the platform and are randomized to the intervention arm will be provided with one pre-paid smartphone with voice and data services for the duration of their study participation. Provision of a second phone in the event of loss or theft will be evaluated on a case-by-case basis.

### Who will take informed consent? {26a}

All informed consent discussions will be completed by a site-delegated study staff member, such as a research coordinator or research assistant.

### Additional consent provisions for collection and use of participant data and biological specimens {26b}

As per institutional guidelines, participants will be asked to complete a “Consent to Communicate by Email” form prior to completing the informed consent discussion. Participants will be asked permission to share their de-identified trial data in an open access database, in line with Canadian Institutes of Health Research (CIHR) best practice. This trial does not involve collecting biological specimens for storage.

## Interventions

### Explanation for the choice of comparators {6b}

Utilizing the NICE COVID-19 treatment guideline, we have selected a comparator group that will be able to answer the question “What is the clinical effectiveness of different service models of multimodality/multidisciplinary post-COVID-19 syndrome rehabilitation in improving patient-reported outcomes (such as quality of life)?” The comparator group will receive usual care and health coaching as required. We chose to compare this to health coaching plus electronic case management in the intervention group as this addresses one of the recommended research questions in the NICE guidelines “What is the clinical effectiveness of different service models of multimodality/multidisciplinary post-COVID-19 syndrome rehabilitation in improving patient-reported outcomes (such as quality of life)?”

### Intervention description {11a}

#### Control arm: health coaching plus usual care (HC)

##### Assessment

Participants randomized to the control arm will be offered assessment by a clinician, guided by the WHO Post COVID-19 CRF (https://www.who.int/publications/i/item/global-covid-19-clinical-platform-case-report-form-(crf)-for-post-covid-conditions-(post-covid-19-crf-)). This is a clinical tool developed by the WHO to guide and document the sequelae of COVID-19 and to ensure that clinical and rehabilitation needs are identified.

##### Investigation

Clinician judgment will be used to decide on further testing needed for clinical care.

##### Management

Control participants will receive a rehabilitation plan developed with their health professional that will be implemented in the 8 weeks after their initial consultation (baseline visit) and access to a personal health coach. The implementation will involve face to face or virtual care from a registered health professional provided by the clinic or research staff. This may be a combination of, but not limited to, occupational therapy, physical therapy, and/or social work/counselling services. The frequency of treatment visits will depend on the individual treatment plan after assessment. All participants will receive access to a health coach.

#### Experimental arm: electronic case management plus usual care

Participants randomized to the experimental arm will receive assessment, investigation and management as outlined above plus access to an electronic case management platform – NexJ Connected Wellness (https://nexjhealth.com/), supported by a personal health coach and which complements the rehabilitation plan. This would include, for example, setting targets for activity that would be monitored with NexJ; educational materials; and, support with medication adherence by reminders. The NexJ platform will include the following sections: Case Plans with Educational Workbooks, Trusted Educational Content (Health Library), Symptom Tracking, Goal Setting, and Reporting.

#### Timing of sessions

All participants will receive health coaching sessions up to once a week. Participants who are using NexJ will have assigned content between sessions. Due to the heterogeneous nature of Long COVID presentation, timing of clinical rehabilitation sessions will be individualized based on the treatment plan. This information will be recorded for each participant.

### Criteria for discontinuing or modifying allocated interventions {11b}

Decisions about modifying individual rehabilitation care or health coaching will be made in collaboration with the most responsible healthcare provider and the Principal Investigator. No predefined criteria will be used with respect to modifying individual interventions.

The trial will be stopped if more than 25% of participants in the intervention group experience at least a 20% increase in severity in their WHODAS sum score. There will be no stopping criteria for benefit given the short duration of the study.

### Strategies to improve adherence to interventions {11c}

No particular strategy will be implemented to improve adherence to the intervention as this is an outcome of the study. As part of the process evaluation, we are examining factors affecting adherence to the intervention. We will evaluate any differences in adherence between the treatment and intervention groups.

### Relevant concomitant care permitted or prohibited during the trial {11d}

As outlined in Table 1, only other trials providing treatment for Long COVID will be prohibited. All other concomitant care is permitted and will be documented.

### Provisions for post-trial care {30}

In the event of a study-related injury or illness, participants will be provided with appropriate medical treatment and care. Financial compensation for lost wages, disability, or discomfort due to an injury or illness is not generally available. After study completion, participants will not have access to the NexJ Connected Wellness platform as this is not publicly available. The participants’ most responsible care provider will ensure a transition back to or continuation of standard care, as required.

### Outcomes {12}

#### Primary outcome

To evaluate the primary outcome, improvement in quality of life, we will use the total sum score of the WHO Disability Assessment Scale (WHODAS 2.0) self-assessment. The WHODAS is a 36-item self-report questionnaire measuring health and disability from the previous 30 days across six domains of functioning: cognition, mobility, self-care, getting along, life activities, and participation. Responses are scored on a 5-point Likert scale: None [0], Mild [1], Moderate [2], Severe [3], and Extreme/Cannot Do [4] [32].

The WHODAS 2.0 has been validated using a cross-cultural approach in 19 countries and within 4 subgroups (general population, populations with physical problems, populations with mental or emotional problems, and populations with problems related to substance use). The WHODAS 2.0 showed excellent overall test-retest reliability (ICC=0.98) and internal consistency (Cronbach’s alpha = 0.98). The survey also showed good cross-cultural sensitivity and concurrent validity [32].

#### Secondary outcomes

##### Long COVID characteristics

To describe the population and their experience with Long COVID, we will administer the WHO Post COVID Case Report Form (CRF) Modules 1, 2, and 3 at baseline and only Module’s 2 and 3 at 12 weeks [33]. The CRF Module 1 captures demographics, pregnancy, pre-COVID health status, and details about the acute COVID infection. CRF Modules 2 and 3 collect vaccination status, occupational status, functioning, post-COVID symptoms, clinical tests, and scales (including neurological, radiographic, blood tests, heart and lung function, mental health, function, and musculoskeletal tests), new diagnoses or complications related to COVID infection, health service use, and symptoms [33]. The CRF will be completed using health record review whenever possible, with missing information completed by interview with the participant.

Currently, little is known about the long-term consequences of infection with COVID-19. In response to this, the WHO has created the WHO Post COVID Case Report Form, which will allow health care providers internationally to collect information on COVID-19 systematically. The use of this CRF will allow for the trial dataset to be aligned with the global WHO Clinical Platform and contribute to expanding our knowledge on Post-COVID-19 condition(s).

All other secondary outcome measures are described in Table 3.

**Table 3:** Secondary outcomes

Measure	Description	Properties
Cognitive Testing	Executive function, visual attention, task switching	The Oral Trail Making Test (O-TMT) (versions A and B) will be used to evaluate executive function, visual attention, and task switching in participants. During O-TMT A, participants will be asked to count out loud from 1 to 25 and is primarily a measure of processing speed and simple attention. During O-TMT B, participants will be asked to alternate saying numbers and letters (1-A, 2-B, etc.) and is a measure of mental flexibility. Time to completion as well as total errors are recorded [34, 35]. The use of time and error rate led to slightly improved classification rates for cognitive impairments compared to either alone [36].
	Verbal learning and memory	The Hopkins Verbal Learning Test-Revised (HVLT-R) will be used to evaluate verbal learning and memory capabilities in participants [37]. The HVLT consists of a 12-item word list, composed of four words from each of three semantic categories. The list is read to participants at a rate of approximately 2 words per second. Participants are then asked to free recall the list of words. This is repeated for 3 trials. After the third trial and a 20–25-min delay (with no forewarning), participants will again be asked to free recall the 12 words and then will be read 24 words and asked to say “yes” after each word that appeared on the recall list (12 targets) and “no” after each word that did not (12 distractors) [38]. The HVLT is available in 6 forms to reduce test-retest learning effects, takes no more than 10 min to administer and is shown to be well tolerated by participants [37].
	Attention, Working memory	The Digit Span subset, a component of the Working Memory Index of the Weschler’s Adult Intelligence Scale - 4th Edition (WAIS-IV), will be used to assess attention and working memory [39]. Participants will be read a series of numbers and then asked to recall the numbers to the examiner in order (forward span), in reverse order (backward span), and in sequence (sequence span). Participants will complete spans of increasing length, with two trials at each length, until an error is made on both trials of a given length. Digit Span has shown good internal consistency and test-retest reliability across age groups [39, 40].
	Verbal fluency	The Phonemic and Semantic Verbal Fluency (or Controlled Oral Word Association Test [COWAT]) test will be used to assess verbal fluency. Participants will be given 1 min to produce as many unique words as possible: (1) within a semantic category (category fluency for Animals); and (2) starting with a given letter (letter fluency for letters F, A, and S). The COWAT has been shown to have sensitivity to brain lesions and conditions with impaired cognition (e.g., traumatic brain injury). Population norms are available for the COWAT for a broad age (16–95) and education (0–21 years) range [41, 42].
PHQ-9	Depression	A 9-item questionnaire that assesses the severity of depression symptoms experienced within the last 2 weeks. Participants are asked to rate each symptom of depression on a Likert scale from 0 (not at all) to 3 (nearly every day), with total scores ranging from 0 (minimal depression) to 27 (severe depression). The PHQ-9 has strong methodological properties with an internal consistency of 0.89 and strong test-retest reliability [43].
GAD-7	Anxiety	A 7-item questionnaire that assesses the severity of anxiety symptoms experienced within the last 2 weeks. The initial validation study, conducted by Spitzer et al. (2006), demonstrated high internal consistency (*α*=0.92) and test-retest reliability (intraclass correlation = 0.83) [44].
PSQI	Sleep	The Pittsburgh Sleep Quality Index (PSQI) is a self-report questionnaire that assesses sleep quality in the previous month and takes 5–10 min to complete. It has been translated into over 50 languages. Using a threshold score of greater than 5, the PSQI showed a diagnostic sensitivity of 89.6% and specificity of 86.5% (kappa = 0.75, *p* < 0.001) in distinguishing “good” and “poor” sleepers [45].
PSTD Checklist 5 (PCL-5)	Post-traumatic stress disorder	A 20-item self-report measure that assesses the presence and severity of PTSD symptoms, corresponding with DSM-5 criteria for PTSD. The PTSD Checklist 5 (PCL-5) scores exhibit strong internal consistency (*α* = .94), test-retest reliability (*r* = .82), and convergent (rs = .74 to .85) and discriminant (rs = .31 to .60) validity [46].
EQ-5D-5L	Quality of life	This is a 5-item questionnaire that assesses health-related quality of life, including mobility, self-care, ability to participate in one’s usual activities, pain or discomfort, and anxiety or depression and a visual analog scale (VAS) which asks participants to evaluate their overall health on a scale from 0 to 100. It is available in multiple languages, and there are standardized scores for patient populations in different settings. The EQ-5D-5L is able to define a unique health state based on the responses to each of the five dimensions of health described above [47].
AUDIT	Alcohol use	The AUDIT questionnaire is designed to assess alcohol consumption, drinking behavior, adverse reactions, and alcohol-related problems. Among those who were known to misuse alcohol, the AUDIT successfully detected an alcohol use disorder 99% of the time [48].
ASSIST	Alcohol and substance use	The ASSIST is a brief interview collecting information regarding use of tobacco, alcohol, cannabis, cocaine, amphetamine type stimulants, sedatives, hallucinogens, inhalants, opioids, and other drugs. Internal Consistency (Chronbach’s alpha) was over 0.80 for the majority of domains and good concurrent validity [49, 50].
FSS	Fatigue	The Fatigue Severity Scale (FSS) is a 9-item self-report that measures the severity and functional impact of fatigue. The scale demonstrated good internal consistency (Cronbach’s *α* 0.88), as well good test-retest reliability and sensitivity to clinical change [51].
Fatigue Numeric Rating Scale	Fatigue	A single-item patient-reported outcome, scored on a scale of 0–10, with good test-retest reliability (ICC=0.79, *p* < 0.008) and sensitivity to change [52].
Pain Numeric Rating Scale	Pain	The Pain Numeric Rating Scale will be used to assess pain intensity using a 0–10 ranking scale, where 0 represents “no pain” and 10 “unbearable pain.” The scale shows good test-retest reliability (ICC=0.72 for single item), and sensitivity [53]. Pain may be categorized using Mild (scores ≤5), Moderate (scores 6–7), or Severe (scores ≥8) [54].
MRC Dyspnoea Scale	Breathlessness	A 5-item self-report scale that evaluates statements of perceived breathlessness. The scale has good correlation to level of disability due to breathlessness [55].
WEMWBS	Wellbeing	The Warwick-Edinburgh Mental Wellbeing Scale (WEMWBS) (short version) is a 7-item scale which measures multiple aspects of mental wellbeing [56]. It has been validated in general population samples and in English and French [57], and is sensitive to change [58].

#### Predictors and potential confounders

At the first assessment, we will collect information on predictors and potential confounders as listed in Table 4.

**Table 4:** Predictors, potential confounders and effect modifiers

Predictors and potential confounders	
Demographic details	At baseline, we will collect information on ethnicity, gender identity, education, income, employment, housing, and living circumstances
Past medical history	This will include body mass index and past medical and mental health disorders
Impact of COVID-19 infection	For those who tested positive, we will record details of the severity and impact of the COVID-19 infection including neurological symptoms such as anosmia and headaches, length of time self-isolating, and time off work
IEQ-SF	The Injustice Experience Questionnaire (IEQ) is a 12-item self-report that assesses perceived injustice associated with injury. The IEQ showed good correlation with measures of catastrophic thinking (*r*=.75, *p* <.01), fear of movement/re-injury (*r*=.58, *p*<.01), depression (*r*=.66, *p*<.01), and pain severity (*r*=.54, *p*<.01).The IEQ also demonstrated good test-retest reliability [59].
Adverse childhood events questionnaire (ACE)	The ACE-Q is a 17-item self-report questionnaire assessing types of adverse exposures during childhood including psychological, physical, and sexual abuse as well as household dysfunction. The ACE questionnaire study found strong relationships between childhood exposure and disease conditions, health risk factors, and a strong dose-response relationship between number of exposures and risk factors for death [60].

#### Process evaluation

To inform the implementation of the intervention beyond the clinical trial and describe the intervention process, we will use the RE-AIM framework [31]. We will assess Reach by recording what proportion of eligible patients participate in the trial and comparing characteristics of participants to non-participants. Effectiveness will be assessed in the clinical trial and separately stratified by sex. We will also record dropouts by sex and severity. We will conduct qualitative interviews with participants to better understand the reasons for participating, the experience of using the NexJ app, and any reasons for drop out. We will use a thematic analysis and aim to complete at least 20 user interviews and will continue until we achieve data saturation. We will gather usage data from NexJ. Adoption at the setting level will be assessed by comparing uptake in the different clinics supplemented by qualitative interviews with clinical staff. Adoption at the staff level will compare high referrers to the study to low referrers and doing qualitative interviews with staff in each group. Implementation will be assessed with a study specific-adherence scale. Maintenance at an individual and setting level will be assessed using the Consolidated Framework for Implementation Research (CFIR) to inform qualitative interviews with key stakeholders to identify barriers and facilitators to introducing NexJ in outpatient clinics.

#### Economic evaluation

We will conduct a cost-utility analysis of an electronic case management system compared with usual care from a perspective of the publicly funded health care system. Costs and outcomes will be assessed within the follow-up period of the trial. Resources and associated costs required to set up and operate a unique instance of the NexJ platform will be estimated from the budget review and staff interview. We will collect health care utilization data, such as hospitalization, emergency department visits, physician visits, prescriptions, and the efficacy of the intervention, from the concurrent trials. We will derive health utility value from the EQ-5D-5L using the Canadian validated algorithm [61], Quality-Adjusted Life Years (QALYs) will be estimated using the total area under the curve method [62]. Unit costs for each health care resource will be obtained from Canadian Sources. Costs will be expressed in 2021 Canadian Dollars.

### Participant timeline {13}

Administration will occur as per the Time and Events Schedule in Table 5. Clinical visits will occur as needed and will be determined during the initial assessment. Clinical visits are not outlined in the time and event schedule as these are a part of routine care. Self-report measures will be completed at home prior to each study visit using the study Electronic Data Capture System (EDCS). Participants will be able to leave and return to the questionnaires whenever they need to complete them. It is anticipated the first and last visit will take approximately 45 min, while weeks 4 and 8 will take approximately 30 min. Participants will complete the WHODAS first and all responses are mandatory. All other self-report measures will be completed in a structured order.

**Table 5:** Time and events schedule

Measures	Time to complete (minutes)	Administration	Baseline	Week 4	Week 8	Week 12
WHO Post COVID CRF Module 1	Variable	Chart review	**X**			
WHO Post COVID CRF Module 2	Variable	Chart review	**X**			**X**
WHO Post COVID CRF Module 3	Variable	Chart review	**X**			**X**
Demographics	<5	Self-report	**X**			
ACE-Q	5	Self-report	**X**			
Injustice Experience Questionnaire (IEQ-SF)	<5	Self-report	**X**			
WHODAS	5	Self-report	**X**	**X**	**X**	**X**
PHQ-9	<5	Self-report	**X**	**X**	**X**	**X**
GAD-7	<5	Self-report	**X**	**X**	**X**	**X**
PCL-5	5	Self-report	**X**	**X**	**X**	**X**
EQ-5D-5L	<5	Self-report	**X**	**X**	**X**	**X**
AUDIT	<5	Self-report	**X**			**X**
ASSIST	5-10	Interview	**X**			**X**
Fatigue Severity Scale	<5	Self-report	**X**	**X**	**X**	**X**
PSQI	5	Self-report	**X**	**X**	**X**	**X**
Warwick-Edinburgh Mental Wellbeing Scale (WEMWBS)	5	Self-report	**X**			**X**
Pain Numeric Rating Scale	<5	Self-report	**X**	**X**	**X**	**X**
MRC Dyspnoea Scale	<5	Self-report	**X**	**X**	**X**	**X**
Cognition (Trailmaking, Hopkins Verbal Learning, Digit Span, Phonemic/Semantic Verbal Fluency)	30	Interview	**X**			**X**
Qualitative Interview (Optional)	30	Interview				**X**

### Sample size {14}

We have based the sample size calculation on the sum of domain scores of the WHODAS 36, as the recommended assessment for Long COVID patients [33]. At the time of writing this protocol, there were no published scores of the WHODAS 36 in people with Long COVID. Therefore, we have based our sample size calculation on WHODAS 36 scores from 25 randomly selected patients attending an existing clinic for Long COVID at TOH run by OWN. These patients had a mean sum of domain scores of 17 (SD=4). The minimally important clinical difference on the WHODAS 36 is about a 10% change in scores [32]. Using these assumptions, we need to recruit at least 126 participants (63 in each group) to have 80% power to detect a 10% change in scores assuming an alpha of 0.05. Allowing for 20% drop out would mean a recruitment target of 152 (76 in each group) people.

### Recruitment {15}

**Fig. 1:**
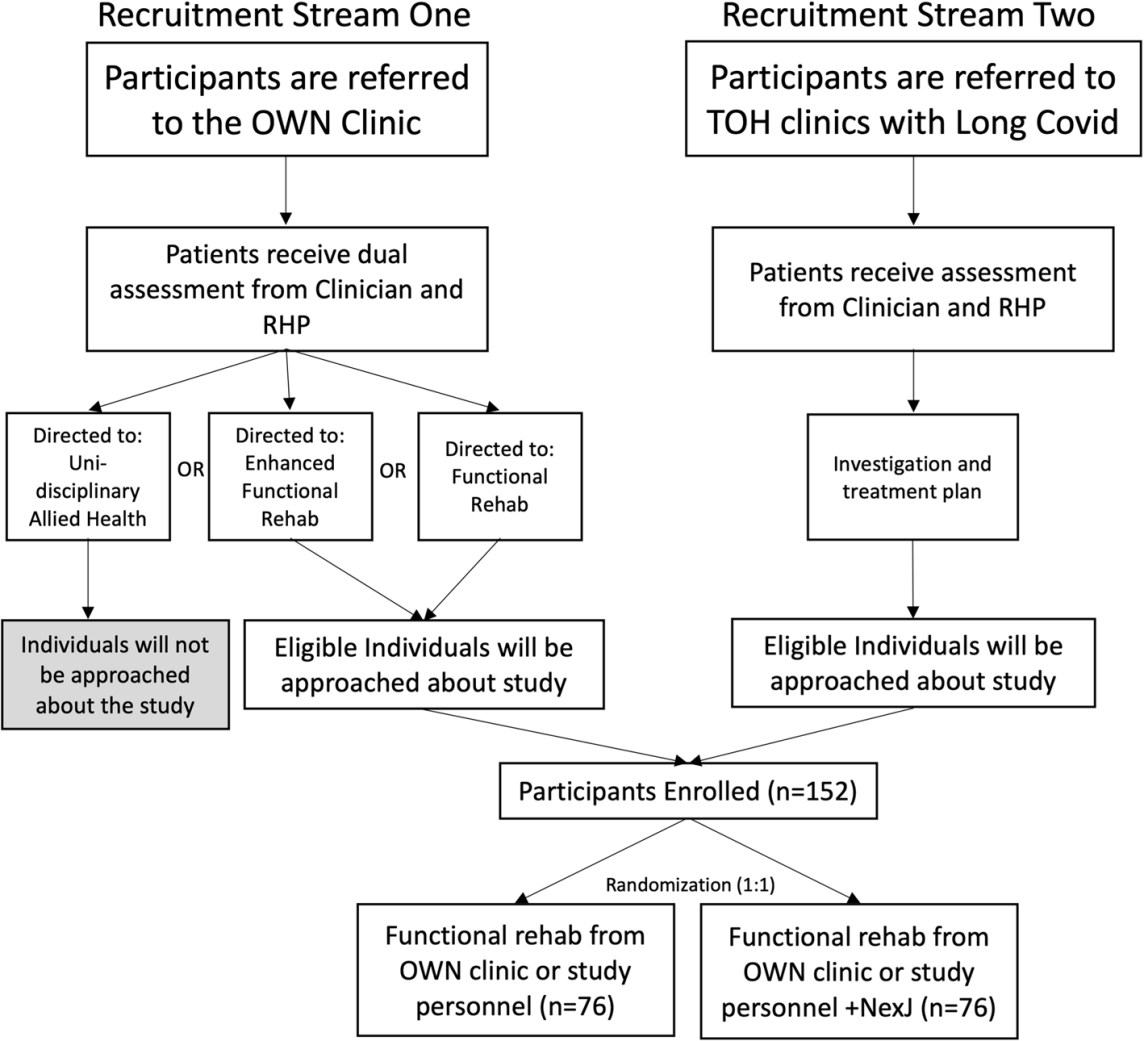
Patient flow

Participants will be recruited at TOH through two potential recruitment streams. The first clinic sees Ontario workers who were infected by SARS-CoV-2 at their worksite, who have made a claim to the Ontario WSIB and are assessed by the OWN based at TOH. The second stream is any clinics within TOH which are seeing patients with Long COVID. These are either clinics specifically for people with Long COVID, for example within respirology, infectious diseases, or psychiatry. The anticipated flow of recruitment is outlined in Fig. 1 below.

## Assignment of interventions: allocation

### Sequence generation {16a}

Randomization will be done remotely by the Ottawa Methods Centre (OMC) through an electronic randomization system. Participants will be randomized at enrollment by a delegated study staff member. As participants are created in the ECDS, they are automatically randomized. The delegated study staff member will be informed of the allocation at the time of randomization.

Allocation will be a 1:1 ratio into the treatment arms. Participants will be stratified by sex and duration of symptoms. The reasons for stratification by sex are that the mortality rate in males is higher than in females and that outcomes may be affected by gendered occupations [63–66]. We will stratify by the duration of symptoms into two groups: (1) those who at enrolment have had symptoms since the onset of their index illness for 12 to 23 weeks, and (2) those who have had symptoms at enrolment for 24 weeks or longer, which is consistent with the classification of Long COVID in the NICE guidelines. We are stratifying for duration of illness as this is one of the main determinants of outcomes.

### Concealment mechanism {16b}

Not applicable. Since the interventions are assigned through random computer generation, allocations are not visible in advance, and no concealment is required.

### Implementation {16c}

Once participants have consented and completed screening determining eligibility, they will be registered in the ECDS and randomization system by the delegated research staff member. Once the participant is registered, the system will automatically allocate the participant to the intervention.

## Assignment of interventions: blinding

### Who will be blinded {17a}

Due to the nature of the intervention, a behavioral intervention where both patient and clinician must use an electronic platform, it is not possible to blind either group to their allocation. Data analysts and manuscript writers will be blind to the identity of the two groups which will be referred to as group A and group B. Once initial manuscript preparation is complete, the groups will be unblinded. We will also assess potential bias in recruitment during an interim analysis.

### Procedure for unblinding if needed {17b}

Not applicable—all participants and treating clinicians are unblinded to the intervention.

## Data collection and management

### Plans for assessment and collection of outcomes {18a}

All self-reported outcome measures will be administered via the EDCS for all study visits. Interview measures, when done remotely, will be completed using a videoconferencing platform. Information from the WHO Post-COVID Assessment CRF will be entered by delegated study staff into the EDCS.

Cognitive assessments will be administered by a trained research staff member. The delegated study staff member will receive direct training from an experienced Psychologist, including rehearsal and independent evaluation, prior to administration with participants.

### Plans to promote participant retention and complete follow-up {18b}

A major difficulty in all clinical research is ensuring participants attend their visits and stay enrolled in the study. Efforts will be made to minimize the time between being approached about the study and the baseline visit. We anticipate lost to follow-up to be no more than 20%. While there is currently no available literature on the lost to follow-up rates for Long COVID rehabilitation, the case management platform we are using has demonstrated significantly higher levels of retention in difficult to treat populations [24]. For those participants in the intervention arm, daily and weekly reminders will be programmed into the NexJ Connected Wellness platform to encourage regular use and increase connectedness with the platform, increasing retention.

We will implement an escalated approach if contact is lost with individuals between study visits. The appropriate response will be determined in collaboration with the Principal Investigator, particularly if there is a potential safety concern. If contact is made with the participant and they are no longer interested in participating, the delegated study staff member will make every effort to ascertain why they are withdrawing their consent. Participants will be sent the link to complete their self-report questionnaires at each time point unless they have withdrawn their consent for participation.

### Data management {19}

All study outcome data will be directly entered into an EDCS. Data will be automatically coded, and the code will be described using a data dictionary. Range checks will be included for illogical entries. Missing items will be flagged for self-entry; however, participants can bypass the warning. A delegated study staff member will follow-up on the missing entry to confirm if the question was missed or the participant is refusing to answer.

The EDCS is a username- and password-protected web-based system. All network, server security and privacy settings are regularly tested and comply with Health Canada recommendations and Good Clinical Practice (GCP) for secured data management services [67, 68]. The EDCS is hosted on a physical server, not in the cloud, that is located at TOH data center in a secure server room with access limited to authorized personnel behind lock doors. The web/database server is behind the TOH firewalls. The OHRI website is secured with the highest rating from Entrust SSL virtual test. The data transfer between the client and server is protected with Entrust SSL 256-bit encryption. All data transfers between the client computers/browsers and the server/database are encrypted via https.

### Confidentiality {27}

All records will automatically be assigned a unique Participant ID during randomization. The Participant ID will be recorded on a master linking log. The master linking log will be password-protected and stored with limited access as per institutional policy. The Participant ID will appear on all documentation included in a participant’s research chart, including study forms and correspondence, in order to maintain participant confidentiality. All correspondence with participants will be de-identified, removing names and other potentially identifying information, prior to being included in the study chart. Some documentation will remain identifiable including the Informed Consent Form, the Participant Information Form, and the Emergency Contact Form. These will not be stored with documentation identified with a Participant ID. Hard copy documentation will be stored in a double locked area (e.g., locked cabinet and secure office space). Study documents will be password-protected, and a log is kept separately from the documents so that access to the files is not lost in the event of staff turnover or during the archiving period. Electronic documents will not be stored on mobile devices (such as a USB or directly on a laptop).

Documents requiring de-identification must use an approved method. Electronic documents must be redacted so that the information removed cannot be recovered at a later time utilizing a tool such as Adobe Pro “Redact” feature, permanently obscuring the data. Hardcopy documentation will be redacted using only a “china marker,” which completely obscures the data. Sharpie, pen, and other writing tools are not permitted as they do not permanently obscure/destroy the data.

Participants’ study information will not be released unless a delegated study staff member obtains written permission from the participant or where required by law. Participants will not be identified in study presentations or publications. Once all study activities are complete, all study records and data exports will be archived at a secure storage facility for a period of 10 years, as per the applicable Network of Networks (N2) Standard Operating Procedure (SOP) and GCP regulations. The 10-year period will begin from the date the last informed consent form is signed.

### Plans for collection, laboratory evaluation, and storage of biological specimens for genetic or molecular analysis in this trial/future use {33}

Not applicable. There are currently no ancillary trials planned.

## Statistical methods

### Statistical methods for primary and secondary outcomes {20a}

The OMC will complete all data analyses. Baseline characteristics will be presented as means (or medians) with associated measures of variation for continuous variables, or frequencies and proportions for categorical data. We will use an intention-to-treat analysis based on all randomized participants. Additionally, we will analyze the primary and secondary outcomes based on the treatment received. Analyses will utilize a Gender-Based Analysis Plus (GBA+) focused Health Equity Impact Assessment (HEIA) [69] approach to examine how sex and gender interact with socioeconomic status, age, race/ethnicity, and rural/urban residence.

Analysis of primary and secondary outcomes will use relative risks and mean differences and be calculated and presented with 95% Confidence Intervals (CI). We will include subgroup analyses of participants by age, sex, and severity of Long COVID. The effects of group (control vs. experimental), Long COVID severity, and adverse childhood events on the linear change over time in the primary outcome (measured at 4 time points) will be analyzed using hierarchical regression. Bivariate associations between primary and secondary outcomes (and among secondary outcomes) measured at baseline and at the end of the study will be estimated with Pearson correlation coefficients (or Spearman correlation coefficients for non-parametric data).

#### Economic evaluation

Results of a cost-utility analysis will be presented as incremental cost per QALY gained. The incremental cost and incremental outcome will be estimated using generalized linear models that explicitly allow for the modelling of normal and non-normal distributional forms of data. The incremental cost-effectiveness ratio will be obtained through the difference in the mean costs of the two strategies divided by the difference in the mean QALYs for each strategy as denoted by the coefficient of the intervention indicator variables. Uncertainty in the analysis will be addressed by estimating 95% CIs using a non-parametric bootstrapping method. For this study, we will obtain 10,000 estimates of costs and outcomes for each strategy. Results from the bootstrapping exercise will also be used to show cost-effectiveness acceptability curves, which represent the probability of NexJ being cost-effective over a range of willingness to pay values that the health system may be willing to pay for an additional unit of QALY.

### Interim analyses {21b}

We will do a health equity impact assessment at 6 months to ensure the study has addressed potential health inequities in recruitment, treatment, and assessment using the Ontario HEIA tool [69].

### Methods for additional analyses (e.g., subgroup analyses) {20b}

Not applicable. There are currently no planned subgroup analyses.

### Methods in analysis to handle protocol non-adherence and any statistical methods to handle missing data {20c}

Efforts will be made to minimize missing data, although it is possible that some amount of missing data may occur. We will examine the pattern of missing data by comparing characteristics of participants with missing data to those of participants with complete data to examine the assumption of missing at random. In the case of substantial missingness (e.g., >5%), missing covariates and outcomes will be imputed using multiple imputation with *m*=10 imputed data sets prior to analysis.

### Plans to give access to the full protocol, participant-level data, and statistical code {31c}

The Principal Investigator will retain ownership of the dataset through the 10-year archiving period. The protocol will be made available open access and will be published, at a minimum, on an open science platform such as Open Science Framework or medRxiv. The anonymized data set will be shared on an open science platform, such as Open Science Framework or Zenodo. The anonymized dataset may also be shared with the WHO to facilitate global post-COVID-19 research and treatment efforts.

## Oversight and monitoring

### Composition of the coordinating center and trial steering committee {5d}

**Table:** 

Committee	Responsibilities	Membership
Trial Steering Committee	Approval of the final protocol; reviewing and advising on progress of study; facilitation of study conduct; end point evaluation; manuscript coordination; knowledge translation	Investigators as listed, other members as outlined in the Trial Steering Committee charter.
Trial Management Committee (Coordinating Centre responsibilities)	Protocol management; study documentation; ethics, contracts; study planning; day-to-day activities; budgets, monitoring; data verification; randomization; support study registered health professionals; investigator support for trial activities; liaise with platform service provider; organize steering committee meetings; implement steering committee action items	Principal Investigator, co-Principal Investigator, Clinical Research Program Manager, Clinical Research Coordinator, Clinical Research Assistant

### Composition of the data monitoring committee, its role and reporting structure {21a}

An independent Data and Safety Monitoring Committee (DSMC) has been convened to assess the progress of the clinical trial, the integrity of the data, and the safety of all participants and to provide recommendations to the Principal Investigator(s). The members of the DSMC serve in an individual capacity and provide their expertise and recommendations. The DSMC will review cumulative study data to evaluate safety, study conduct, scientific validity, and data integrity of the study. A charter will outline the full responsibilities of the DSMC. At a minimum, the general responsibilities of the DSMC will be:

- To evaluate any reports of safety-related events in the study to ensure the ongoing safety of participants;
- To consider factors external to the study when relevant information becomes available, such as scientific or therapeutic developments that may have an impact on the safety of the participants or the ethics of the study;
- To review the conduct of the study;
- To make recommendations to continue, modify, or terminate the study.

Membership will be comprised of four independent, interdisciplinary members. Possible fields of expertise include statistics/biostatistics, epidemiology, methodology, psychiatry, and the ethics of clinical trials. Members will be identified in the DSMC charter.

### Adverse event reporting and harms {22}

Participant wellbeing and safety will be monitored by the Investigators and Registered Health Professionals (RHPs), within the restrictions of their respective professional colleges. Adverse events will be collected at each study visit. SOPs will be developed for escalation of participant safety concerns, for example by setting a threshold increase on mental health scales where the Principal Investigator, or appropriate most responsible care provider, is notified for follow-up. If a significant worsening of symptoms occurs, the individual notified or Principal Investigator will follow-up with the participant and will provide intervention as necessary. Reporting of adverse events and serious adverse events will follow institutional procedures as laid out in SOPs and Research Ethics Board (REB) guidance.

Participants may find some questions in the questionnaires uncomfortable. Participants may refuse to answer any questions to which they do not want to respond. Expected adverse events include worsening fatigue, decline in symptoms reported at baseline related to the study intervention, and eye strain/fatigue from using the smartphone application.

### Frequency and plans for auditing trial conduct {23}

An internal study monitor will be selected prior to the start of the trial. The monitor will not be involved in data collection activities and will be one step removed from the clinical trial. Before the launch of recruitment, the internal monitor will ensure that appropriate training has been completed and documented for all delegated study staff.

After enrollment of the first participant, the internal study monitor will review all documentation for this participant to ensure any errors or challenges are identified and resolved before being carried through subsequent participants. Throughout the duration of the study, the internal monitor will perform monitoring visits on a minimum quarterly basis, or more frequently as needed based on recruitment rates. An internal monitoring plan will guide the process and materials to be reviewed at each visit, as well as follow-up procedures.

At the end of study, the monitor will lead the study close out activities in collaboration with the study staff to ensure that all study records and essential documentation are complete and ready for analysis, REB closure, and archiving.

Throughout the study, the OHRI Clinical Facilitators may also audit the study. These audits may be planned or unplanned. If an audit is requested, the study team will assist the OHRI in completing their review of the study. If the internal monitor discovers significant or repeated issues in their monitoring visits, they may request the assistance of the OHRI Clinical Facilitators for their recommendations.

### Plans for communicating important protocol amendments to relevant parties (e.g., trial participants, ethical committees) {25}

Any subsequent modifications to the study protocol, including changes to study objectives, study design, patient population, sample sizes, study procedures, or significant administrative changes will be agreed upon by the Investigators and submitted to the REB of record for review and approval prior to implementation. Participants will be informed in writing of any modifications or new information that may impact their willingness to participate in the study. If required, participants may also be asked to sign a Consent Update Form, as approved by the REB.

## Dissemination plans {31a}

We plan to take a diverse approach to our knowledge mobilization and dissemination plan. We have engaged the Public Health Agency of Canada (PHAC) as Knowledge Users in our study. PHAC will work with the investigator team to identify relevant targets of knowledge exchange activities within the federal health portfolio, as well as with national and international stakeholders. Primary outcomes publications refer to any presentation or publication that presents data on the primary outcome measure as detailed in this protocol. The primary outcome publication will be submitted within 6 months of the completion of data collection. The primary outcomes publication will be open access and any secondary publications will be made open access whenever possible.

All study results will be released to study participants, the general medical community, the general public, and other identified stakeholders. Other forms of dissemination include academic publications, conference presentations, stakeholder meetings, and presentations to the general public. Lay knowledge products will be designed in collaboration with lived experience partners to ensure that dissemination is impactful and relevant to the community.

## Discussion

There is currently little evidence about the optimal treatment of Long COVID patients or the use of digital health platforms in this population. There are currently no systematic reviews of treatment for Long COVID and much of the existing literature is considered low-quality evidence. Important operational issues relate to the privacy and confidentiality of any information stored on the app; however, the study team has ensured safeguards are in place to minimize this risk. Additionally, navigating virtual visits and fluctuating lockdowns poses variable challenges throughout the duration of the study. The protocol has been designed to be delivered hybrid or fully virtual, if required, to seamlessly accommodate any changes in access to in-person resources. The results of this trial could result in rapid, scalable, and personalized care for people with Long COVID which will decrease morbidity after an acute infection. Results from this study will also inform decision making in Long COVID and treatment guidelines at provincial and national levels.

### Trial status

This study received initial approval by the OHSN-REB in September 2021, with an amended protocol approved (protocol version date 23 Feb 2022). Recruitment was opened in May 2022. At the time of writing this manuscript, one participant had been enrolled. The estimated completion date for primary data collection is May 2023.

## Data Availability

The Principal Investigator will retain ownership of the dataset through the 10-year archiving period. Once the primary outcomes manuscript is published, the anonymized data set will be shared on an open access platform, such as Open Science Framework or Zenodo. Prior to this, any data required to support the protocol can be supplied upon request to the Principal Investigator (SH).

## Abbreviations

ACE-Q: Adverse Childhood Events Questionnaires
ASSIST: Alcohol, Smoking and Substance Interview Screening Test
AUDIT: Alcohol Use Disorders Identification Test
CI: Confidence Interval
CIHR: Canadian Institutes of Health Research
COWAT: Controlled Oral Word Association Test
CRF: Case Report Form
DSMC: Data and Safety Monitoring Committee
ECM: Electronic Case Management
EDCS: Electronic Data Capture System
FNRS: Fatigue Numeric Rating Scale
FSS: Fatigue Severity Scale
GAD-7: Generalized Anxiety Disorder 7-item scale
GBA+: Gender-Based Analysis Plus
HEIA: Health Equity Impact Assessment
HVLT-R: Hopkins Verbal Learning Test-Revised
GCP: Good Clinical Practice
IEQ-SF: Injustice Experience Questionnaire – Short Form
N2: Network of Networks
NICE: National Institute for Healthcare and Excellence
OHRI: Ottawa Hospital Research Institute
OHSN-REB: Ottawa Health Sciences Network Research Ethics Board
OMC: Ottawa Methods Centre
OWN: Ontario Workers Network
PCL-5: PTSD Checklist-5
PCR: Polymerase chain reaction
PHAC: Public Health Agency of Canada
PHQ-9: Patient Health Questionnaire 9-item scale
PNRS: Pain Numeric Rating Scale
PSQI: Pittsburgh Sleep Quality Index
QALY: Quality-adjusted life years
RAT: Rapid Antigen Test
REB: Research Ethics Board
RHP: Registered Health Professional
SOP: Standard Operating Procedure
O-TMT: Oral Trail Making Test
TOH: The Ottawa Hospital
WAIS-IV: Weschler’s Adult Intelligence Scale - 4th Edition
WEMWBS: Warwick-Edinburgh Mental Wellbeing Scale
WHO: World Health Organization
WHODAS: WHO Disability Assessment Scale
WSIB: Workplace Safety and Insurance Board
